# Notes and News

**Published:** 1887-12-10

**Authors:** 


### NOTES AND NEWS.

COLLECTED AND COMMUNICATED.

ON Friday evening a musical and literary entertainment in aid of the funds of the Ballymena Cottage Hospital was given in the local Protestant Hall. The concert was excellent, and the audience large.

IT is matter for regret, and will no doubt cause some otherwise preventible suffering, that the main buildings of the Brompton Hospital for Consumption are now entirely closed and must remain closed for some time, owing to the necessity of extensive alterations and improvements in connexion with the sanitary arrangements. With a public spirit which does them credit, and for which they have always been remarkable, the committee propose to send to convalescent homes on the south coast, at the expense of the hospital, such patients as may have improved under the treatment they have received, and as would further benefit by a change to the seaside. That old and tried friend of the hospital, Mr. Dobbin (the secretary) will gratefully receive and acknowledge any subscriptions and donations which may be sent to him. In such a case as this, those who have and to spare should not stand upon the order of their giving, but give at once. Very many, no doubt, will do this gladly, for the Consumption Hospital, Brompton, is an institution of which everybody is proud, and with which everyone is, we have reason to believe (*mirabile dictu*), satisfied also.

THE following vacancies are announced this week, the amount of salary in each case being given after the name of the institution :—

**Resident Medical Officers.**

Ashton-under-Lyne Infirmary. House Surgeon.—£80.
Royal Westminster Ophthalmic Hospital, King William Street, Strand. House Surgeon.
Sheffield Public Hospital and Dispensary. Junior House Surgeon. —£50.
Miller Hospital and Royal Kent Dispensary, Greenwich. Junior Medical Officer.—£30.
Southport Infirmary and Dispensary. House Surgeon, to visit out-patients.—£100.

**Non-resident Medical Officers.**

St. George's Hospital. Assistant Physician.
St. George's Hospital. Assistant Surgeon.
Hull Royal Infirmary. Honorary Dental Surgeon.

**Matron.**

Batley and District Cottage Hospital.—£50.

**Nurses.**

Great Yarmouth Temporary Hospital. Night Nurse.—£20, and washing.
Dewsbury District Infirmary, Dewsbury. Charge Nurse.—£24.
Glasgow Sick Poor and Private Nursing Association. Trained Medical and Surgical Nurses.
Isle of Wight Nursing Institution, Ryde. Medical and Surgical; also Monthly.
Royal Albert Hospital, Devonport. Certified Trained Nurse. —£25, with uniform.
Workhouse Infirmary Nursing Association, 44, Berners Street. Several.
Home for Nurses, Cambridge. Monthly, Medical and Surgical. Apply Lady Superintendent.

THE Mayor of Salford (Mr. Alderman Dickens) presided at the sixtieth annual meeting of the subscribers of the Salford and Pendleton Royal Hospital on Tuesday morning. Among those present were : Dr. Dixon Mann, Mr. Alderman Nuttall, Mr. N. Shelmerdine, J.P., and Mr. F. Moss, J.P. The report stated that a considerable portion of the new building had been opened for general use, and that provision is now made for 116 beds. The finances of the charity are in a satisfactory condition, except as regards annual subscriptions, and in this there appears a falling-off for the year. It may be hoped that, there being no Jubilee next year, the annual subscriptions, not only to the Salford Hospital, but to very many other institutions, which also report a falling-off, will revive again.

A LECTURE, under the auspices of the Edinburgh Health Society, was delivered in the Free Assembly Hall, Edinburgh, by Mr. Arthur W. Hare, F.R.C.S., on "The Channels of Infection, and Hints for Avoidance of Disease." Professor Sir Douglas Maclagan presided, and the lecture was listened to with interest by a large audience.

THE Special Supplement issued with last week's number, showing how work can be provided for the unemployed, has attracted a good deal of attention and discussion. We propose to show next week how each London constituency can effectually solve the question of adequately providing for its own distress, and for the unemployed who reside within its boundaries. Meanwhile, those who desire to have copies of the Special Supplement issued with the last number should make early application to the publisher, 2, Paternoster Square, E.C.

THE Lady Ada Scott, the Lady Charlotte Home, Mrs. Darling, the Misses Campbell Swinton, Kimmerghame, and Miss St. Clair, with many others, took part in a sale of useful and fancy work held on behalf of the proposed Cottage Hospital at Coldstream, on Friday. The sum of nearly a thousand pounds has already been raised on behalf of this excellent Border project.

HER Royal Highness Princess Mary Adelaide, with the Princess Victoria of Teck and her Royal Highness Princess Frederica, were present on Wednesday at the third annual distribution of the work done by Associates of the Surrey Needlework Guild, which took place at The Gables, Surbiton, by invitation of Mrs. Wilberforce Bryant. Upwards of eight thousand useful garments were sent to various hospitals, homes, and poor parishes.

WHEN a young lady has been elevated by popular vote to the proud position of belle of the town or district in which she lives, there are never wanting old friends who try to subdue the vanity she may naturally feel, by telling her that she is not half so pretty as her mother was, and cannot compare with her grandmother for loveliness. In a somewhat similar strain Dr. George Harley has been assuring us that we conceited moderns are very much inferior to our forefathers of the stone age. He admits that we weigh more, are taller and stronger, and live longer than our remote ancestors —this all the statistics of anthropology go to prove ; but as a set-off to these comforting assurances we are told that neolithic man surpassed his descendants in the power of recovering after bodily injury. This advantage, the desirability of which we cannot dispute, he owed to his inferior development of nervous system. The less highly developed an animal is, the greater is its recuperative, and even regenerative, power. A crab can replace a lost claw, and many of the lowest animals can suffer being cut into several parts without injury —even with apparent advantage ; for each part becomes a a new animal, and the amœboid population can thus be indefinitely increased. Among barbarous nations the same recuperative power exists, though in an infinitely less degree. A savage warrior will recover from injuries that would inevitably kill a European. But if we consider all that nerve-force has done for us—how it, through the medium of what we call brains, has changed the face of the world, increased our resources, given us comforts of which the neolithic man had no conception, and powers that would have seemed to him miraculous ; how with these has come added capacity for enjoyment to balance our added power of suffering ; if we realise that all the machinery of our civilisation and many of our highest sentiments, are the result of that increased nervous development which has lessened our recuperative power ; we will be contented with our own lot, and say that primitive man's facility in recovering from injuries was dear at the price.

We have been obliged to hold over our list of the names of those who have intimated their intention of attending the Conference in February to discuss the scheme, entitled "The Sick, the Churches, and the Hospitals," published on page 91 of No. 58, vol. iii., and several important letters, until next week. We have also in type articles by the Duke of Argyll and others, which will appear shortly.

The Mayor of Manchester (Mr. Alderman J. J. Harwood) presided at the annual meeting of the members of the Hospital Saturday and Sunday Committees Fund, on Wednesday. Among those present were Sir Henry Roscoe, M.P., Mr. C. E. Schwann, M.P., Mr. Oliver Heywood, Mr. Alderman King, Mr. Joseph Broome, the Mayor of Salford, and the Rev. J. Davenport Kelly. According to the report of the secretary (the Rev. J. Henn) the amount received from the Sunday collections was £4,603 18*s*. 7*d*., which is very nearly the same as the amount collected last year, whilst the Saturday Fund realized £3,135 11*s*. 9*d*., or nearly £200 more than in 1886. Unlike London, the amount collected on Hospital Saturday in Manchester is almost equal to that collected on Sunday, but the Sunday Fund itself is miserably small, being only about a-tenth of that collected in London.

Consumption, like poverty, is always with us; therefore, the account given by M. Julius Kercher of what he considers to be a specific for that disease will be read with interest, even though not with belief. He is the owner of a large ultramarine factory, where great quantities of sulphur are used daily, causing a great production of sulphuric acid vapour; and he states that during the forty years the factory has been in existence not one of his workmen had died from consumption, and some who had exhibited symptoms of the disease before entering his service became cured in a few weeks. He accounts for this by the theory that the sulphur vapour destroys the bacteria in the lung that cause the disease, and suggests that in many cases, especially where the disease is not too far advanced, the inhalation of sulphur fumes would tend to recovery.

In Madame Lind-Goldschmidt the Worcester Infirmary has lost a generous and constant friend. At a recent meeting of the executive committee, under the presidency of the Rev. W. H. R. Longhurst, a resolution was passed in the following terms: "That the Executive Committee of the Worcester General Infirmary, gratefully remembering the generous services rendered to this institution by Madame Jenny Lind-Goldschmidt, desire to place on record their deep sense of the loss which the whole community has sustained in her lamented death, and to assure Mr. Goldschmidt and the members of his family of their sincere sympathy with them in their bereavement." The Chairman said it was very difficult to choose words which conveyed quite adequately their feelings; but he hoped it would be considered that the resolution did express with a certain measure of correctness what that committee desired to record. Madame Jenny Lind-Goldschmidt's death had been very widely and very deservedly lamented, and he could not help thinking it was a matter of honour to them that this institution should be so closely associated with the name of one who had set such a bright example, who had used the splendid gifts with which she was so richly endowed so unselfishly and for the good of others, and who had taken such a wide and general interest in works of charity, more especially in that particular form of charity with which that institution was associated. He at any rate felt quite sure that the Governors of that Institution would never cease to revere the memory of one who had shown such practical sympathy with the sick and suffering of the city and neighbourhood.

Small-pox has broken out at Leicester, five cases, all in the same family, having occurred. The origin of the outbreak has not been discovered. Four of the five patients had been vaccinated, and in all these the attack is said to have been slight. The last patient—an unvaccinated person—had the disease in a confluent form.

Speaking at Bournemouth the other night, Canon Wilberforce said total abstinence had the power of prolonging more lives than all the doctors in England, and of saving more money than all the savings banks in the United Kingdom, and also of preventing more crime than all the police in the metropolis. He said old admirals and generals had come out nobly as special policemen when the country was in danger. There was now a call for special constables to fight the national sin—drunkenness.

The thirteenth anniversary dinner of the Metropolitan Dairymen's Benevolent Institution was held at the Freemasons' Tavern on the 1st inst., Mr. J. Welford being in the chair. Among the guests were Mr. Aird, M.P., who, in replying to the toast of "The House of Commons," expressed his fellow-feeling in some aspects of the hard-working lives of milkmen. It had been his lot often, he said, after an unprofitable night spent in the House, to return home "with the milk." The Chairman explained the objects of the Association, and made an appeal for subscriptions, which was liberally responded to, the Secretary reading out a list the total of which amounted to nearly £1,100.

The conflict between man and the bacillus anthracis has been going on in Bradford. A man who had been engaged in sorting Persian wool was attacked with wool-sorter's disease. The case becoming serious, the patient was taken to the hospital to submit to treatment by hot-air baths. It was thought that if the patient's temperature could be raised to 107 degrees the bacilli which cause the disease would be killed. So far the experiment was successful; the bacilli were destroyed, but the patient's brain was in such an unfavourable condition that he died soon after of collapse. In spite of the fatal result, however, there is still a belief in the district that the treatment adopted in this case would have been successful had it been employed at an earlier stage of the disease. It would certainly be cause for gratitude if anything could be done, either by precaution or cure, to protect the men who in this and other trades are exposed to such subtle and ineradicable diseases in the pursuit of their calling.

Example is better than precept, saith the copy-book, and a certain wise Board of Guardians have shown themselves able to profit by the old saying in a matter of sanitation. In consequence of an outbreak of fever it was necessary that every house in a particular village should be washed with hot lime; but the contractors who sent in estimates for the work asked too much both of time and money for it for their proposals to be accepted. An officer of the union who had some knowledge of human nature volunteered to get the whitewashing done both quickly and cheaply. He ordered all out-door paupers to produce within a week a paper certifying that their houses had been whitewashed outside and in; the materials for the work to be furnished by the union. In a day or two the two old soldiers entrusted with the distribution of lime, pails, and brushes complained that more of these were wanted than the numbers of paupers could account for. "Keep your eyes shut," said the official, "and hand out the materials to any extent asked for." The inhabitants generally were borrowing whitewash and brushes from the paupers, with the result that within a week the village was thoroughly whitewashed at the cost to the ratepayers of only the materials, and to the satisfaction of everyone interested in the matter. May the diplomatic official live for ever!

